# Studies on Cell Compatibility, Antibacterial Behavior, and Zeta Potential of Ag-Containing Polydopamine-Coated Bioactive Glass-Ceramic

**DOI:** 10.3390/ma12030500

**Published:** 2019-02-06

**Authors:** Rocío Tejido-Rastrilla, Sara Ferraris, Wolfgang H. Goldmann, Alina Grünewald, Rainer Detsch, Giovanni Baldi, Silvia Spriano, Aldo R. Boccaccini

**Affiliations:** 1 Institute of Biomaterials, University of Erlangen-Nuremberg, 91058 Erlangen, Germany; rocio.tejido@fau.de (R.T.-R.); alina.gruenewald@fau.de (A.G.); rainer.detsch@fau.de (R.D.); 2 Colorobbia Consulting s.r.l., 50053 Sovigliana Vinci, Florence, Italy; 3 Department of Applied Science and Technology, Politecnico di Torino, 10129 Torino, Italy; sara.ferraris@polito.it (S.F.); silvia.spriano@polito.it (S.S.); 4 Centre for Medical Physics and Technology, University of Erlangen-Nuremberg, 91052 Erlangen, Germany; wgoldmann@biomed.uni-erlangen.de

**Keywords:** polydopamine, silver, antibacterial, biocompatibility, bioactive glass-ceramic, coatings

## Abstract

Dopamine is a small molecule that mimics the adhesive component (L-DOPA) of marine mussels with a catecholamine structure. Dopamine can spontaneously polymerize to form polydopamine (PDA) in a mild basic environment. PDA binds, in principle, to all types of surfaces and offers a platform for post-modification of surfaces. In this work, a novel Ag-containing polydopamine coating has been developed for the functionalization of bioactive glass-ceramics. In order to study the interactions between the surface of uncoated and coated samples and the environment, we have measured the surface zeta potential. Results confirmed that PDA can interact with the substrate through different chemical groups. A strongly negative surface zeta potential was measured, which is desirable for biocompatibility. The dual function of the material, namely the capability to exhibit bioactive behavior while being antibacterial and not harmful to mammalian cells, was assessed. The biocompatibility of the samples with MG-63 (osteoblast-like) cells was determined, as well as the antibacterial behavior against Gram-positive *Staphylococcus carnosus* and Gram-negative *Escherichia coli* bacteria. During cell biology tests, uncoated and PDA-coated samples showed biocompatibility, while cell viability on Ag-containing PDA-coated samples was reduced. On the other hand, antibacterial tests confirmed the strong antimicrobial properties of Ag-containing PDA-coated samples, although tailoring of the silver release will be necessary to modulate the dual effect of PDA and silver.

## 1. Introduction

The average life expectancy of humans is increasing worldwide thanks to advances in medicine and science. As a consequence, there has been an increase in osteoarthritis and other pathologies in elderly people, who consequently require orthopedic and dental implants [1]. The introduction of implants has the intrinsic risk of microbial infection, which can lead to implant failure. Bacterial infections are becoming more difficult to treat due to antimicrobial resistance (AMR). In the European Union, which has a population of ~500 million people, there are about 25,000 deaths per year due to bacterial infections [2]. Not only does this represent an unacceptable loss of lives, it carries an economic burden; if AMR is not controlled, the annual global gross domestic product will lose 3.8% by 2050 [3]. New approaches must therefore be put forward to prevent bacterial colonization of different surfaces, thus limiting the spread of infections. 

Numerous biomaterials have been developed over the years for the application as orthopedic or dental implants, including stainless steel 316 L, cobalt-based alloys, titanium and its alloys, polymers, glass-ceramics and bioactive glasses, and their composites [1]. Among these, bioactive glasses and glass-ceramics have received much attention, because of their desirable mechanical properties and their capability to interact with hard [4,5] and soft tissues [6]. 

Surface functionalization offers the possibility to tailor the properties of a material’s surface to obtain an optimal interface between the substrate and the biological environment [7]. Since the discovery of the outstanding properties of polydopamine [8], several materials have been coated with this oligomer, which mimics the molecules found near the plaque–substrate interface of mussels [8,9,10]. Thanks to the presence of catechol, amine, and imine moieties in its structure, polydopamine can undergo further functionalization by electroless metallization with copper or silver [11,12]. Silver nanoparticles and nanocomposites are well known for their strong antibacterial capabilities [13,14]. Silver has been also shown to be non-cytotoxic to human cells [13,14,15]. 

The incorporation of silver in bioactive glasses has been investigated for many years [16,17,18]. This is usually achieved by the sol-gel technique; another option is to coat the surfaces with silver using different techniques such as plasma spraying, molten salt ion exchange, or sputtering [16,17,19,20]. 

Coatings, based on polydopamine incorporating silver particles, represent an attractive approach to obtain dual-functional composite layers on the surface of biomaterials. Such composites should facilitate interactions with biological tissues without showing cytotoxicity and also exhibit antibacterial capability [21,22,23,24,25]. In our previous study [26], Ag-containing polydopamine coatings on bioactive glass-ceramic surfaces were developed; however, only a limited characterization of the coatings has been reported. In this work, such coatings have been characterized by testing their biocompatibility. We have carried out antibacterial tests against Gram-positive *Staphylococcus carnosus* and Gram-negative *Escherichia coli* bacterial strains. We have further conducted studies regarding the surface zeta potential of the samples and hypothesize that due to the presence of several chemical moieties, polydopamine can bind differently to different biomaterials depending on the characteristics of the substrate. 

## 2. Materials and Methods

### 2.1. Fabrication of the Samples and Structural Characterization

All tests were performed on sintered glass-ceramic (labelled BGC1) pellets, uncoated and coated with PDA and PDA@Ag (BGC1@PDA, BGC1@PDA@Ag), of which the details are described elsewhere [26]. Briefly, we produced a melt-derived glass of the nominal composition BGC1 (in wt%): 47.8% SiO_2_, 4.9% Na_2_O, 0.4% K_2_O, 30.6% CaO, 2.9% MgO, 11.8% P_2_O_5_, and 1.6% CaF_2_. Ground BGC1 powder was then cold-pressed at 0.7 MPa for 30 s to form cylindrical pellets with a diameter of 11 mm and a height of 3 mm. The pellets were sintered at 860 °C for 30 min. In order to coat the pellets with polydopamine, sintered pellets were immersed for 24 h in aqueous solution of dopamine hydrochloride (Sigma-Aldrich, Schnelldorf, Germany) in Tris-buffer solution (Tris(hydroxymethyl) aminomethane, Sigma-Aldrich, Schnelldorf, Germany). The solution’s pH value was adjusted to pH 8.5 using HCl 1 M. The pellets were then thoroughly rinsed with deionized water. Additionally, the PDA-coated pellets (BGC1@PDA) were further functionalized by immersing them for 24 h in an aqueous solution of AgNO_3_ 5 × 10^−3^ M (Sigma-Aldrich, Schnelldorf, Germany). BGC1@PDA@Ag pellets were then rinsed with deionized water and allowed to dry in normal air.

The surface roughness was measured for each type of coating by using a laser profilometer (UBM Microfocus Expert, ISC-2). A measurement length of 5 mm was used with a scanning velocity of 400 points per second. The mean roughness (R_a_) and maximum roughness (R_max_) were calculated using the LMT Surface View UBM software (UBM Messtechnik GmbH, Ettlingen, Germany). R_a_ is calculated as the average roughness of the coating’s surfaces by measuring microscopic peaks and valleys, while R_max_ is calculated by measuring the vertical distance from the highest peak to the lowest valley. Three samples were measured for each type of coating, and mean values of R_a_ and R_max_ were reported with standard deviation.

Morphological analysis of the samples, prior to and after coating, was performed by means of scanning electron microscopy (SEM) (Auriga SEM instrument Zeiss, Oberkochen, Germany). Samples were sputter-coated with gold in vacuum. 

### 2.2. Surface Zeta Potential

The zeta potential of the samples was measured by means of the streaming potential technique, using an electrokinetic analyser (SurPASS, Anton Paar, Graz, Austria) equipped with an adjustable gap cell. All measurements were performed in simulated body fluid (SBF) [27], which was diluted in ultrapure water up to a pH of about 7.4 and had a conductivity close to 16 mS·m^−1^ (measured by the monitoring function of the instrument), without pH titration during measurements. For the analyses, two cylindrical samples (11 mm diameter, 3 mm height) were prepared for each type (bare BGC1, BGC1@PDA and BGC1@PDA@Ag) and mounted parallel to each other on the sample holder of the cell. The gap was adjusted close to 100 μm and the electrolyte flow to approximately 100 mL·min^−1^. The zeta potential values were obtained from measured streaming currents using the Helmholtz–Smoluchowski Equation (1):

(1) $$\zeta =\frac{\mathrm{dU}}{\mathrm{dp}}\times \frac{\eta }{\epsilon \times \epsilon_{0}}\times \kappa_{B},$$

where dU/dp is the slope of streaming potential vs. differential pressure, η is electrolyte viscosity, ε is dielectric coefficient of electrolyte, ε_0_ is permittivity, and κ_B_ is electrolyte conductivity.

### 2.3. Preconditioning of the Samples

Uncoated BGC1 pellets were sterilized by dry heat (160 °C for 2 h). The samples were placed in a 24-well plate and covered with 1 mL DMEM (+10% FBS, +1% penicillin/streptomycin (PS)). The pH of the medium was measured after 1, 2, 3, 4, 7, and 8 days. Measurements were made in duplicate and the average was calculated. After the measurements, the medium was removed and fresh medium was added. The preconditioning of the samples was carried out under sterile conditions.

### 2.4. Cell Biology Studies

#### 2.4.1. Cell Seeding and Culture

MG-63 (osteoblast-like) cells (Sigma-Aldrich, Schnelldorf, Germany) were used. The cells were cultured at 37 °C in an atmosphere of 95% humidified air and 5% CO_2_, in Dulbecco’s modified Eagle’s medium (DMEM; Gibco, ThermoFisher Scientific, Waltham, MA, USA) supplemented with 10 vol % fetal bovine serum (FBS; Gibco, Germany) and 1% of penicillin/streptomycin (PS; Gibco, ThermoFisher Scientific, Waltham, MA, USA). Cells were grown to 80% confluence in 75 cm^2^ culture flasks, washed with phosphate buffered saline and detached using trypsin/EDTA (Sigma-Aldrich, Schnelldorf, Germany). Cells were counted by a hemocytometer (Neubauer improved) and diluted with culture medium to a final concentration of 1 × 10^5^ cells/mL. Subsequently, 1 mL of cell solution was seeded in direct contact in a 24-well cell culture plate. To ensure statistical significance, eight replicates of each sample type were performed. 

#### 2.4.2. Cell Viability

The viability of MG-63 cells was assessed using the WST-8 assay kit (Sigma-Aldrich, Schnelldorf, Germany). WST-8 (2-(2-methoxy-4-nitrophenyl)-3-(4-nitrophenyl)-5-(2,4-disulfophenyl)-2H-tetrazolium, monosodium salt) is reduced by cellular dehydrogenase to a formazan product, which is directly proportional to the number of living cells. 

After 48 h of incubation, the culture medium was completely removed, and samples were washed with PBS. Subsequently, 0.25 mL of WST-8 medium (containing 1 vol % of WST-8 reagent and 99 vol % of DMEM medium) was added and incubated for 2 h. Afterwards, 100 μL of supernatant from each sample was transferred into a well of a 96-well plate, and the absorbance at 450 nm was measured with a microplate reader (PHOmo, Autobio Labtec Instruments Co. Ltd., Zhengzhou, China). From the obtained absorbance, the cell viability was calculated by taking the absorbance of each specimen (A_i_) and of the respective control (A_0_) as follows:

(2) $$\mathrm{Cell}\;\mathrm{viability}\left(\%\right)=\frac{A_{i}}{A_{0}}\times 100,$$

#### 2.4.3. Cell Morphology

To visualize the adhered cells on the samples, green Calcein AM (calcein acetoxymethyl ester, Invitrogen, ThermoFisher Scientific, Waltham, MA, USA) cell-labelling solution was used to stain the cytoplasm of the cells. Cell culture medium was removed and 0.25 mL of staining solution containing 0.5 vol % of dye-labelling solution and 99.5 vol % of PBS was added to the samples and incubated for 30 min. Afterwards, the solution was removed and the samples were washed with 0.5 mL PBS. Cells on the surface were fixed by 3.7 vol % paraformaldehyde. Samples were washed with PBS again and blue fluorescent DAPI (4′,6-diamidino-2-phenylindole dihydrochloride, Roche, Basel, Switzerland) was added to stain the nucleus. The samples were incubated for 5 min, and the solution was removed. The samples were left in PBS for microscopic viewing, using a fluorescence microscope (Axio Scope, ZEISS, Jena, Germany).

### 2.5. Statistical Analysis

The differences in analytical parameters between the different samples were analyzed by one-way analysis of variance (ANOVA). The significance level was set as * *p* < 0.05, ** *p* < 0.01 and *** *p* < 0.001. For comparison of the mean values, the Tukey post hoc test was used (N = 8).

### 2.6. Antibacterial Tests

A direct contact bacterial assay was performed on uncoated and coated BGC1 pellets. The dimensions of the pellets were 10 mm (diameter) and 2 mm (height). The samples were tested before and after a pre-incubation time of 8 days in DMEM (Dulbecco’s modified Eagle’s medium) to maintain the same conditions as in the case of cell viability tests. 

Prior to coating the samples, they were dry-sterilized in an oven at 160 °C for 2 h. Afterwards, the samples were coated as described elsewhere [26]. After the coating of the samples, they were sterilized by UV irradiation for 1 h. 

Gram-positive *Staphylococcus carnosus* and Gram-negative *Escherichia coli* were chosen as test bacterial strains. Isolated colonies of both Gram-positive and Gram-negative bacteria were suspended in 10 mL of lysogeny broth (LB #968.1, Carl Roth GmbH) and grown overnight in an orbital shaker at 100 rpm at 37 °C. 

The next day, the fresh bacteria suspension was diluted to an optical density of 0.015 at 600 nm (OD600) (Biophotometer Plus, Eppendorf AG, Hamburg, Germany). 

Uncoated and coated samples were placed in a 24 well-plate with 2 mL of LB, and fresh bacteria suspension was inoculated into the samples and put in the incubator at 37 °C. Optical density (OD600) was measured after 1, 4, 8, 24, and 48 hours. To ensure statistical significance, three replicates of each sample type were performed. In addition, bacterial cultures were done in duplicate, on different days. 

## 3. Results and Discussion

### 3.1. Structural Characterization

The surface roughness was measured, and results are summarized in Table 1. It can be seen that for all samples the roughness is quite homogeneous. In this case, the roughness measurements are related to the substrate only, since polydopamine coating has been determined to be ~50 nm in thickness, as reported in literature [9,28]. In this way, for the roughness measurements, the influence of the substrate would be more significant than the polydopamine film itself.

**Figure 1:**
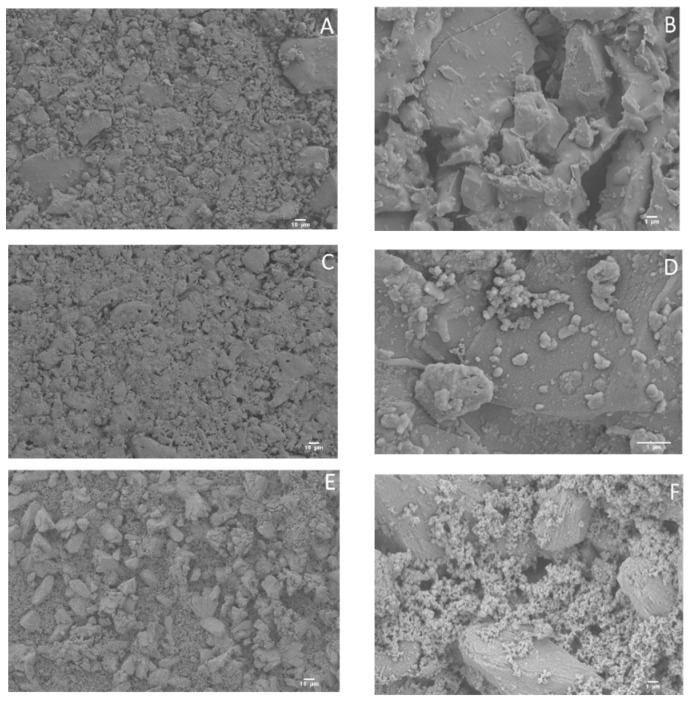
Scanning electron microscopy (SEM) micrographs of uncoated BGC1 (**A**,**B**), BGC1@PDA (**C**,**D**), and BGC1@PDA@Ag (**E**,**F**).

In order to study the morphology of the surface, SEM micrographs were obtained (Figure 1). In the case of BGC1, it is possible to observe sharpened microcrystals (Figure 1A,B), although the overall surface is quite homogeneous. For BGC1@PDA, the surface is smoother, probably due to the deposition of polydopamine (Figure 1C,D), which forms spherical aggregates. After the deposition of silver onto the surface of the coated bioactive glass-ceramic, some silver aggregates are visible, being well distributed on the surface (Figure 1E,F). 

### 3.2. Surface Zeta Potential

The contact between a solid surface and a water-based medium leads to the development of a surface charge at the interface. This charge is one of the surface characteristics, which could affect the interaction between the material and the biological environment (e.g., protein adsorption, cellular, and bacterial adhesion) [29]. In this context, zeta potential measurements were carried out to determine the surface charge at physiological pH (about 7.4). The pH of an aqueous solution is the driving force for an acid-base reaction, meaning that at high pH values, the dissociation of acidic groups will be enhanced while the protonation of basic groups will be suppressed, and vice versa.

The surface charge on polydopamine films is probably due to quinone imine and catechol groups [30]. More specifically, the positive or negative surface charges may arise from the reversible dissociation and deprotonation/protonation of amine and catechol groups, featuring PDA zwitterionicity [10]. It has been reported in the literature that the overall charge of PDA coatings is negative, although there is no general agreement regarding the zeta potential value; values reported vary between −4.58 mV (at pH = 7) and −39 mV (in Tris-buffer at pH = 8.5) [30,31,32,33], which can strongly depend on the measurement conditions (pH, electrolyte). Obtained results on surface zeta potential measurements are summarized in Table 2. 

**Figure 2:**
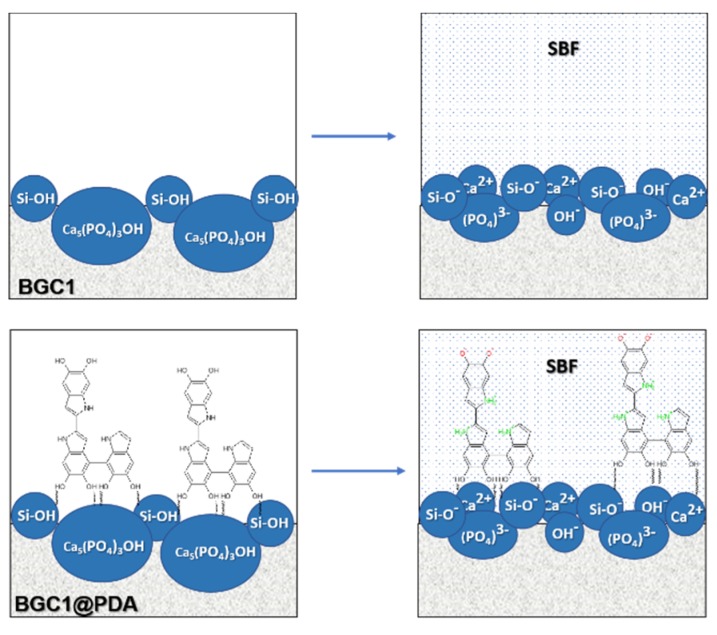
Schematic representations of the origin of negative charge on the uncoated (BGC1) and coated (BGC1@PDA) surface in diluted SBF at pH ≈ 7.4.

For uncoated BGC1, we obtained a highly negative zeta potential value (−120 ± 9 mV). Such negative surface charge at physiological pH is in accordance with the acidic isoelectric point of silica-based bioactive glasses [34]. Moreover, the negative surface charge of BGC1 is likely the result of the presence of siloxane (Si–OH) groups and hydroxyapatite (Ca_5_(PO_4_)_3_(OH)) on the surface (Figure 2), which in the presence of an aqueous solution at pH ~7.4 remains negatively charged. The negative surface charge of the glass-ceramic BGC1 could enhance the affinity and adhesion of cells. It has been reported that strongly negative surface charges promote the adsorption of specific proteins, leading to increased cell adhesion [35]. 

When BGC1 was modified with PDA, the value of the zeta potential at physiological pH remained negative, but its absolute value was reduced (−83 ± 1 mV) with respect to uncoated BGC1. It is possible that, thanks to the presence of PDA, positive ions were attracted from the solution (for example Ca^2+^), increasing the positive charge of the surface; this is in accordance with the literature [36,37,38]. However, this hypothesis is unlikely in the present case because, due to the high dilution of SBF, the availability of Ca^2+^ is relatively low. 

It has been proposed in the literature that polydopamine could interact with aqueous solutions through various mechanisms due to its molecular structure [39]. If PDA binds differently, it means that exposed chemical groups would be different. Therefore, it is possible that for BGC1, polydopamine binds through OH^−^ groups from quinone, leaving more amine groups exposed to the aqueous environment and thus provoking the coated surface to become less negative.

With the introduction of silver onto the surfaces of PDA-modified samples (BGC1@PDA@Ag), the value of the zeta potential becomes slightly more negative (−98 ± 1 mV) than for BGC1@PDA. This behavior could be explained by the presence of silver particles which possess negative zeta potential, as described in the literature for silver nanoparticles [40,41,42] and for coatings containing silver nanoparticles at the investigated pH [43]. 

In summary, the measurements reported here evidence a strong negative charge on all tested surfaces in physiological conditions. The absolute value of these charges can depend on the typology and distribution of the surface functional groups as well as on their acidity strength and on their effect on surface wettability. However, a clear attribution of these differences cannot be obtained by measurements at a fixed pH; indeed, a zeta potential titration as a function of pH should be performed in future works to clarify this point.

### 3.3. Preconditioning of the Samples

**Figure 3:**
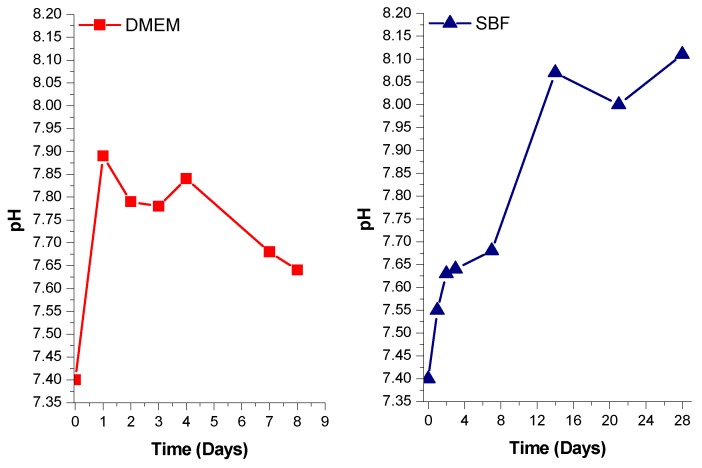
pH variation of DMEM (**left**) and SBF (**right**) containing uncoated BGC1 pellets.

Figure 3 shows the comparison of pH variation in DMEM and in SBF in contact with uncoated BGC1 pellets as a function of time. It must be taken into account that in the case of DMEM, the medium was changed every day, while for SBF the medium was not changed. Both measurements were carried out under static conditions. Observing the behavior in SBF, it is quite clear that a chemical process is occurring at the surface of BGC1 as already proved elsewhere [26]. In this work, the selected medium for pre-treatment was DMEM, since it is the medium used in cell biology tests. The preconditioning time needed for the pH to be lower than 7.75 was selected as 8 days, which is in good agreement with the work of Verné et al. [44]. The limit for the pH value, set to be lower than pH 7.75, has been established as an optimal value for the adhesion of osteoblasts [45]. The duration of the preconditioning treatment, namely 8 days, was relatively short. DMEM does not seem to provoke an extreme reaction at the surface of BGC1. Since BGC1 has a low content of Na_2_O (4.9%), a rapid exchange of sodium ions is not expected, which would cause a detrimental burst of local pH increase. Finally, the morphology of the samples also plays an important role in determining their bioreactivity [46]. It should be noted that in this study we have used BGC1 in the form of dense pellets, which exhibit a slower rate of ion exchange (bioreactivity) compared, for example, with porous materials or powders, which have much higher surface areas. 

### 3.4. Cell Biology Studies

**Figure 4:**
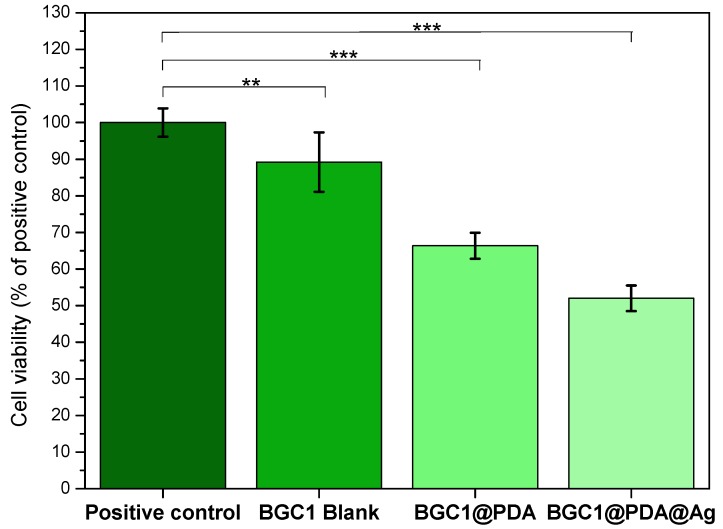
Viability of MG-63 cells cultured onto BGC1, BGC1@PDA and BGC1@PDA@Ag samples for 48 hours. Significant differences are indicated in comparison to control: * *p* < 0.05, ** *p* < 0.01 and *** *p* < 0.001 (Tukey’s posthoc test).

**Figure 5:**
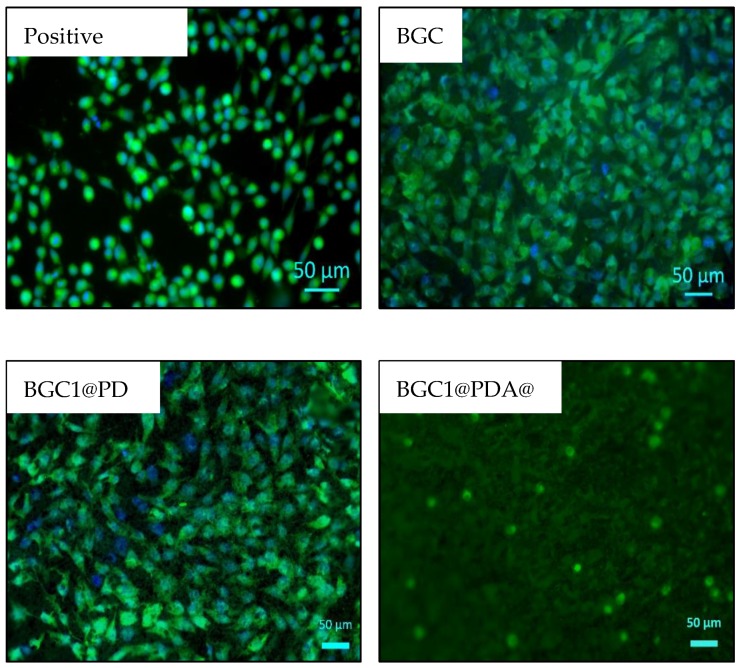
Fluorescence microscope images on different representative samples showing the results of calcein-DAPI staining of MG-63 cells after 48 hours of direct culture.

The viability of MG-63 cells cultured onto BGC1, BGC1@PDA, and BGC1@PDA@Ag samples for 48 hours was determined by WST-8 assay, and the results are shown in Figure 4. The bioactive glass-ceramic BGC1 showed, as expected, good compatibility with mammalian cells. Sintered BGC1 was determined to have a glassy matrix (15.8%) and the following crystalline phases: hydroxyapatite (47.6%), wollastonite (28.7%), and cristobalite (7.9%) [26]. An important phase in BGC1 is wollastonite. This crystalline phase has compatibility with bone tissue, as described in literature [47]. In the same way, hydroxyapatite and glass-ceramic implants have shown favorable interactions with marrow stromal cells [48,49,50]. Figure 5 shows fluorescence microscope images of MG-63 cells after 48 hours of direct culture. For BGC1, it was difficult to determine the morphology of the cells because of the brightness of the surface. However, the surface is seen to be completely covered by cells. 

Polydopamine has been proven to have good cell compatibility, mostly due to (among other properties) its hydrophilicity, stiffness, and surface charge [9,10,51,52]. Figure 4 shows a slight suppression of cell viability for BGC1@PDA, which can be related to a modification of the surface roughness during the coating process. It is important to highlight that there is a strong influence of the substrate, since the polydopamine coating on BGC1 does not form a continuous film, reaching only 50 nm of thickness. Ryu et al. determined that polydopamine surfaces lead to mammalian cell proliferation without toxicity [36]; in addition, Chien and Tsai described that polydopamine does not support the adhesion of all types of cells [53]. On this basis, the lower number of cells on BGC1@PDA samples compared to BGC1 may not be related to cell death, but to a lower rate of proliferation. On the other hand, it can be seen in Figure 5 that cells spread well on BGC1@PDA surfaces and were interconnected to each other, which is an expected behaviour of bioactive materials. 

BGC1@PDA@Ag samples exhibited a strong decrease in cell viability (52%). Fluorescence microscope images (Figure 5) show that cells are round shaped, which indicates cell stress. It is possible to observe calcein-stained cells, which means that cells are alive but under high stress. Forte et al. [24] used polydopamine as an interface between calcium phosphate and silver. It was also found that for certain samples coated with silver, cell toxicity occurred [24]. Therefore, more research efforts should be undertaken to optimize the silver dose to obtain an effective dual-function material.

### 3.5. Antibacterial Test

**Figure 6:**
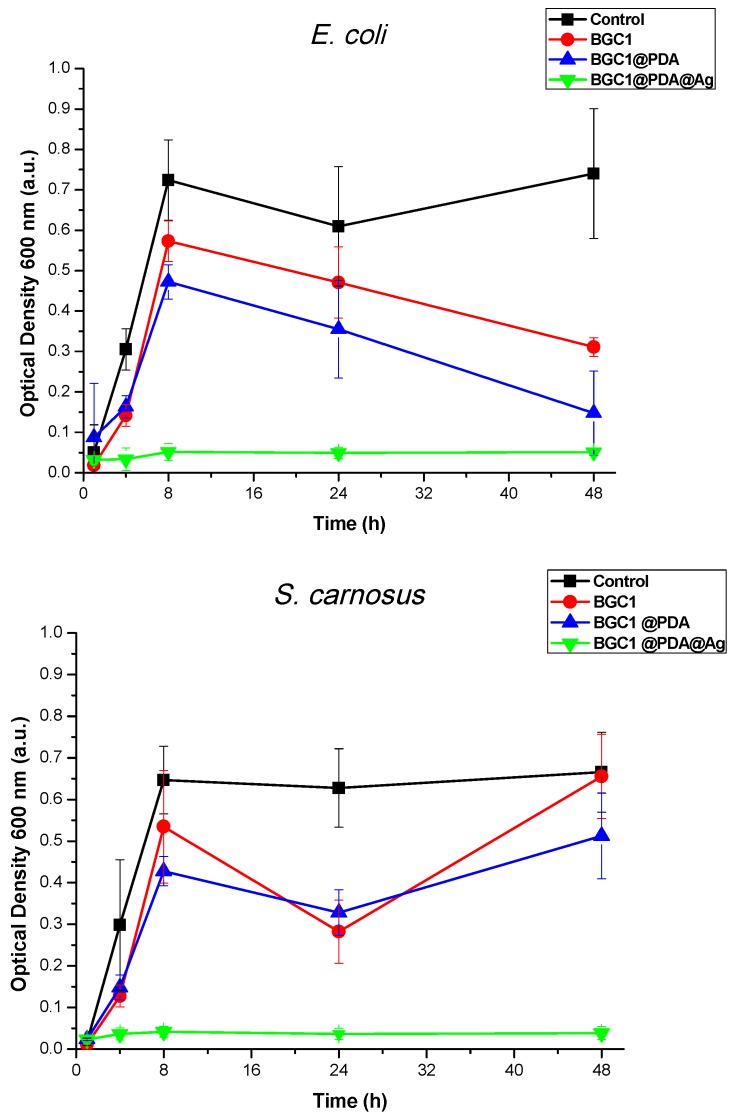
Turbidity measurements on suspensions of both tested *Staphylococcus carnosus* and *Escherichia coli* strains in lysogeny broth (LB) medium on different samples, showing their different antibacterial effects.

Figure 6 shows antibacterial test results against *S. carnosus* (Gram-positive) and *E. coli* (Gram-negative) bacteria strains after 48 hours of incubation in LB medium. For the test carried out against *S. carnosus*, BGC1 and BGC1@PDA did not show a significant effect on the growth of bacteria suspended in the medium. The preconditioning of the samples was important to confirm that there was no increase of local pH for BGC1, because of the lack of any dissolution process within the measuring time period, avoiding bacterial death. In the case of BGC1@PDA, since polydopamine confers hydrophilicity to the samples and does not have an active antimicrobial component, no significant antibacterial effect was observed. This result is in good agreement with the literature, as reports have shown that polydopamine coating itself has no antimicrobial effect against Gram-positive bacteria strains [25]. 

For the test carried out against *E. coli*, it seems that both BGC1 and BGC1@PDA inhibited the growth of bacteria after 24 hours of incubation. There is a controversy in literature, as some authors have described that polydopamine has an intrinsic antimicrobial effect, which is relatively weak against *E. coli* [32,54]. Meanwhile, some other works have determined that polydopamine itself does not show an antibacterial effect against *E. coli* [55]. In Figure 6 it is possible to observe a similar trend for both samples (BGC1 and BGC1@PDA), rejecting the hypothesis that polydopamine itself presents antimicrobial properties. 

The tests carried out on BGC1@PDA@Ag samples against both *S. carnosus* and *E. coli* showed that the material is able to strongly reduce the growth of both chosen bacterial strains, as expected. A similar effect has been reported in the literature, in which several materials have been surface-modified with polydopamine and silver particles [10,21,25,32,56,57].

## 4. Conclusions

A bioactive glass-ceramic, BGC1, and the functionalized samples, BGC1@PDA and BGC1@PDA@Ag, showed a strongly negative surface zeta potential, which is desirable for in vitro biocompatibility. Tests carried out with the MG-63 cell line demonstrated the non-toxicity of BGC1 and BGC1@PDA. BGC1@PDA@Ag showed a moderate biocompatibility. Antibacterial tests indicated that BGC1@PDA@Ag possess a strong antimicrobial effect against both Gram-positive and Gram-negative bacterial strains. An antibacterial effect of PDA was not observed in the present experiments.

These promising findings encourage further investigations, which should lead to the tailoring of silver content in PDA-modified bioactive glass-ceramic to obtain a dual-function composite with strong antibacterial activity and non-toxicity for mammalian cells. 

## Figures and Tables

**Table 1:** Roughness measurements of the different samples investigated.

-	R_a_ (μm)	R_max_ (μm)
Sintered ceramic-glass pellets, uncoated (BGC1)	1.4 ± 0.3	12 ± 5
BGC1 coated with polydopamine (BGC1@PDA)	1.0 ± 0.2	8 ± 2
BGC1 coated with polydopamine and Ag (BGC1@PDA@Ag)	0.8 ± 0.1	6.1 ± 0.3

**Table 2:** Surface zeta potential of the different samples investigated (measurements in simulated body fluid (SBF)).

-	Initial Conditions		Measurements in SBF	
-	pH	Conductivity (mS·m^−1^)	pH	*Z* (mV)
BGC1	7.32	16.14	7.34 ± 0.01	−120 ± 9
BGC1@PDA	7.33	16.84	7.33 ± 0.00	−83 ± 1
BGC1@PDA@Ag	7.38	16.20	7.35 ± 0.00	−98 ± 1
